# Self-Reported Illicit Drug Use Among Norwegian University and College Students. Associations With Age, Gender, and Geography

**DOI:** 10.3389/fpsyt.2020.543507

**Published:** 2020-12-10

**Authors:** Ove Heradstveit, Jens Christoffer Skogen, Marit Edland-Gryt, Morten Hesse, Lotte Vallentin-Holbech, Kari-Jussie Lønning, Børge Sivertsen

**Affiliations:** ^1^Regional Centre for Child and Youth Mental Health and Child Welfare, NORCE Norwegian Research Centre, Bergen, Norway; ^2^Center for Alcohol & Drug Research, Stavanger University Hospital, Stavanger, Norway; ^3^Department of Health Promotion, Norwegian Institute of Public Health, Bergen, Norway; ^4^Department of Public Health, Faculty of Health Sciences, University of Stavanger, Stavanger, Norway; ^5^Department of Alcohol, Tobacco, and Drugs, Norwegian Institute of Public Health, Oslo, Norway; ^6^Centre for Alcohol and Drug Research, School of Business and Social Sciences, Aarhus University, Aarhus, Denmark; ^7^Norwegian Medical Association, Oslo, Norway; ^8^The Student Welfare Association of Oslo and Akershus (SiO), Oslo, Norway; ^9^Department of Research and Innovation, Helse Fonna HF, Haugesund, Norway; ^10^Department of Mental Health, Norwegian University of Science and Technology, Trondheim, Norway

**Keywords:** illicit drug use, university students, college students, gender differences, long-term trends

## Abstract

**Background and Aims:** Several studies have pointed to relatively high levels of illicit drug use among students in higher education compared to the general population. The aim of the present study was to provide an updated examination of self-reported illicit drug use among Norwegian University and college students.

**Methods:** Data stem from the SHoT study (Students' Health and Well-being Study), a nationwide cross-sectional survey for higher education in Norway including Norwegian full-time students aged 18–35. Self-reported illicit drug use across a range of specified drugs comprised the outcome variables. Information on gender, age, and study location (geographical area) was also collected and used as stratification variables. The SHoT-survey from 2018 (*N* = 50,054) was used for the analyses of associations between demographical variables and illicit drug use, while trends in illicit drug use were estimated by comparing the 2018-results with data from the SHoT-surveys conducted in 2010 and 2014.

**Results:** The proportion of students reporting having ever tried illicit drugs increased from 2014 to 2018, for both males (30.8 vs. 36.7%) and females (17.5 vs. 24.0%, both *p* < 0.001), while only minimal changes occurred between 2010 and 2014. The most commonly used illicit drugs during the past 12 months in 2018 were cannabis (15.2%), followed by MDMA (4.0%), cocaine (3.0%), and LSD/psilocybin (2.1%). Illicit drug use showed both linear increase with age, and inverted U-shaped relationships that peaked in the age span from 23 to 28 years of age. Males reported higher illicit drug use compared with females for all drugs. Proportions of illicit drug use varied across geographical areas within the country, with the highest use being reported in the Oslo area (the largest city and capital of Norway).

**Conclusions:** The present study reports an increase from 2010 to 2018 among Norwegian University and college students in the proportion of those reporting to have tried illicit drugs. Despite varying proportions of use across type of drug, age, gender, and geographical location, the overall high levels of illicit drug use past 12 months confirm the need to address illicit drug use in this population.

## Introduction

While alcohol use is widely studied among students in higher education, far less empirical data is available related to illicit drug use in this population (1). Previous research has demonstrated that alcohol use is “alarmingly high” in higher education student populations both in Norway (2, 3) and other Western countries [e.g., (4, 5)]. Several studies have also pointed to a high level of illicit drug use among students in higher education compared with the general young adult population (6–9), including higher rates of cannabis, cocaine, and amphetamine use (1, 10).

Both social and coping motives are described as important drivers of illicit drug use in a range of studies (11–13). Across a range of substances, including cannabis, amphetamine, ecstasy, LSD, and cocaine, common functions of use are relaxation, to get intoxicated/“high,” enhancement of activity, and the alleviation of negative mood (14). For students in particular, the motives may be recreational, related to mood-enhancing or used to increase study performance (15, 16). Stimulants such as Ritalin (methylphenidate) and amphetamine are among the most commonly used drugs in this respect (17). Among Norwegian higher education students, lifetime use of stimulants as a study drug was reported by 2–4% (18). Although a temporary increase in memory may be experienced, the use of these drugs is according to a literature review associated with detrimental negative health effects, while not improving learning and school grades (17).

Previous studies have found that cannabis during the past decades has been the most commonly used illicit drug among higher education students (5, 15, 19, 20), and regular use of cannabis over time is known to be related to severe consequences such as failing to attend to classes, problems with concentration (21), and reductions in motivation (22, 23). Amphetamines and ecstasy were the second and third most commonly used drugs in a Swedish student population in the early 2000s (19) and a more recent UK study reported that amphetamine and LSD were the second and third most commonly drugs used among university students (24). Further, an Italian study reported that cocaine and psychedelic mushrooms were the second and the third most commonly used drugs among university students (20).

Drug trends can change rapidly due to factors such as availability, harm perception and attitudes toward using illicit drugs (25). We need more data to show the trend in illicit drug use among university/college students to inform the development and implementation of appropriate interventions that might help the students to choose a less risky behavior regarding substance use.

An important question in this respect is how rates of illicit drug use vary across age groups. Motives for drug use may potentially differ across the age span (26). In a Swedish University sample, the highest illicit drug use was found among those aged 20–24 years, compared to both younger and older students (19). In comparison, a Norwegian study on illicit drug use among young adults in a nightlife arena (another group characterized by higher substance use than the general population, and which includes a large group of students), reported that the youngest age group (16–20 years) had the highest prevalence of illicit drug use (27). However, it is not evident which age groups within university/college students that display the highest prevalence of use when stratifying by different types of illicit drugs, something that remains to be studied.

Illicit drug use accounts for a significant proportion of the global burden of disease (28). Still, no research exists concerning the distribution of a wider array of illicit drug use among Norwegian University and college students, something which needs to be addressed. Moreover, prevalence rates of illicit drug use tend to vary substantially across countries in the general adolescent (29), adult (30), and student population (31), and rates of drug use vary over time even within a given country [e.g., (32)]. In addition, illicit drug use may vary considerably across geographical areas within a country [e.g., (19)]. It is therefore important to provide a recent update on the status of illicit drug use among higher education students, focusing on differences across larger urban areas (i.e., the capital of Norway, Oslo) and less populated areas in the country.

### Aims

The aim of the present study is to present developments in self-reported illicit drug use across age and gender for Norwegian University students, in addition to differences in geographical areas. First, we will present the gender-specific proportion for having tried any illicit drugs between 2010, 2014, and 2018. Next, we will present frequency of self-reported use for different types of illicit drugs across age and gender groups, as well as age- and gender-adjusted estimates of use across major study locations in Norway using data from 2018.

## Methods

### Study Populations

The SHoT study (Students' Health and Well-being Study) is a national survey first launched in 2010 among students enrolled in higher education in Norway, initiated by the three largest student welfare organizations in Norway [Sammen (Bergen and surrounding area), SiT (Trondheim and surrounding area) and SiO (Oslo and Akershus)]. The SHoT-studies are completely electronic, using web-based questionnaire-platforms and email and SMS invitations. SHoT2018 and previous waves have previously been described in detail (3, 33, 34). Data for the SHoT2010-study was collected during the period 11 October to 8 November 2010 and included 6,053 Norwegian full-time students <35 years of age (response rate: 22.6%). The data for the SHoT2014-study was collected in the period from 24 February 2014 to 27 March 2014 and included 13,525 Norwegian full-time students <35 years of age (response rate: 28.5%). Data for the SHoT2018 study was conducted between 6 February and 5 April 2018, and the entire population of Norwegian full-time students, ≤ 35 years of age enrolled at a higher educational institution (both in Norway and abroad) were invited (*N* = 162,512). In total, 50,054 students completed the questionnaires, yielding a response rate of 30.8%, with 48,818 (97.5%) eligible for the present study.

### Ethics

The SHoT2018 study was approved by the Regional Committee for Medical and Health Research Ethics in Western Norway (no. 2017/1176). Electronic informed consent was obtained after a complete description of the study to the participants. Approvals for conducting the SHoT2010 and SHoT2014 studies were granted by the Data Protection Officer for research at the Norwegian Center for Research Data.

### Age, Gender, and Geographic Location

Age and gender were self-reported: gender (male, female), age (coded as a continuous variable [range: 18–36 years], and an ordinal variable: [18–20, 21–22, 23–25, and 26–35 years]). For age, *N* = 601 (1.23%) reported age 36, and they were included in the age group 26–35 years group. Information about study location was also self-reported. Only major study locations were used for analyses of geographic differences (*N* = 38,052): Oslo area (36.5%), Bergen area (24.9%), Trondheim area (21.6%), Northern Norway (12.0%) and Stavanger area (5.0%). Less populated or more scattered study locations were excluded (*N* = 10,766). However, respondents from these areas were included for other analyses.

### Questions About Drug Use

The introductory question related to illicit drug use was “Have you ever tried other drugs [than alcohol] (e.g., narcotic drugs or prescribed medication with an intoxicating effect)?,” with “yes” and “no” as response options (35). Those responding “yes” were given the question “How often have you used the following drugs during the last 12 months?” followed by a list of 12 specified types of illicit drugs: (1) Amphetamine, methamphetamine, (2) benzodiazepines without prescription (using the most common brand names in Norway, such as Sobril, Valium and the like), (3) ecstasy, (4) gamma-hydroxybutyrate (GHB), (5) heroin, (6) cocaine, (7) LSD, psilocybin, (8) MDMA, (9) Ritalin® without prescription, (10) synthetic cannabinoids (spice), (11) other illegal drugs and (12) cannabis (hash/marihuana). Responses were coded as “never,” “1 time,” “2–4 times,” “5–50 times” and “More than 50 times” for the purposes of this study (35). Also included in the list described above, was a non-existing drug called “relevin,” and endorsement of this was used as an indicator of participants with potentially invalid response patterns, and the responders that indicated use of this drug were omitted from the study. The number of positive responses on this fake drug was negligibly small (0.02%).

### Development in Drug Use (2010, 2014, 2018)

SHoT was conducted in 2010, in 2014 and in 2018. Not all questions are similar across waves, such as use of specific drug types, but use of drugs ever (“Have you ever tried drugs other [than alcohol] (e.g., illicit drugs or prescribed medication with an intoxicating effect)?”) were similar, allowing a comparison across waves. The eligible sample employing data from all three waves consisted of *N* = 69,747 participants. Of them, *N* = 691 had missing information about age, *N* = 197 had missing information about gender, and *N* = 990 had missing information about drug use—with *N* = 1,848 (2.7%) unique individuals being excluded. This left *N* = 67,899 available for analysis of the development in illicit drug use among Norwegian students.

### Statistical Analysis

**Figure 1:**
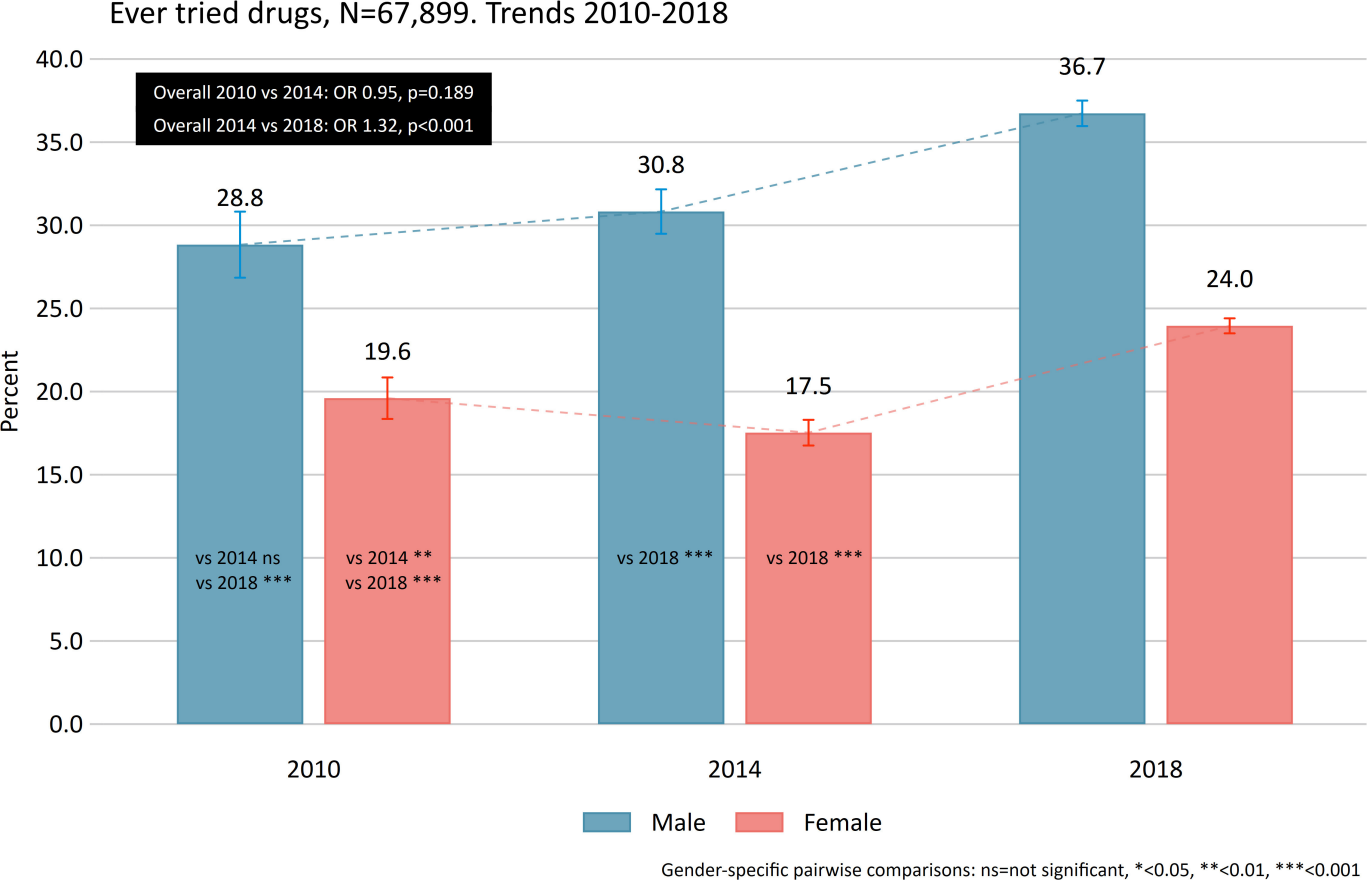
Ever used drugs. Gender-specific age-adjusted proportions between 2010, 2014, and 2014.

First, self-reported age-adjusted proportions of drug use ever across gender was calculated for 2010, 2014, and 2018, along with pairwise comparisons between years using logistic regression models (Figure 1). Estimates are presented as odds ratios (OR).

**Figure 2:**
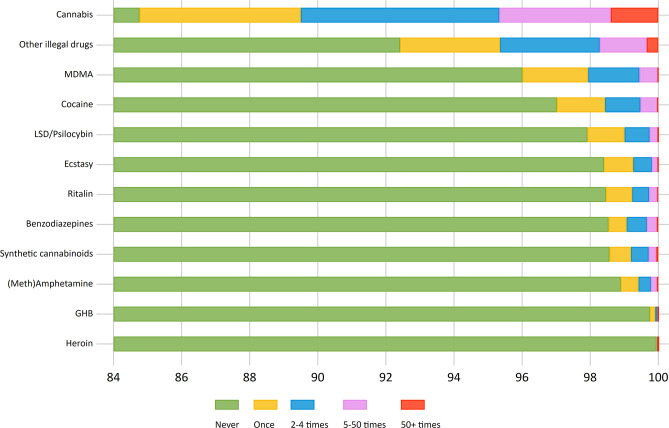
Frequency of last 12-months use across drug types.

**Figure 3:**
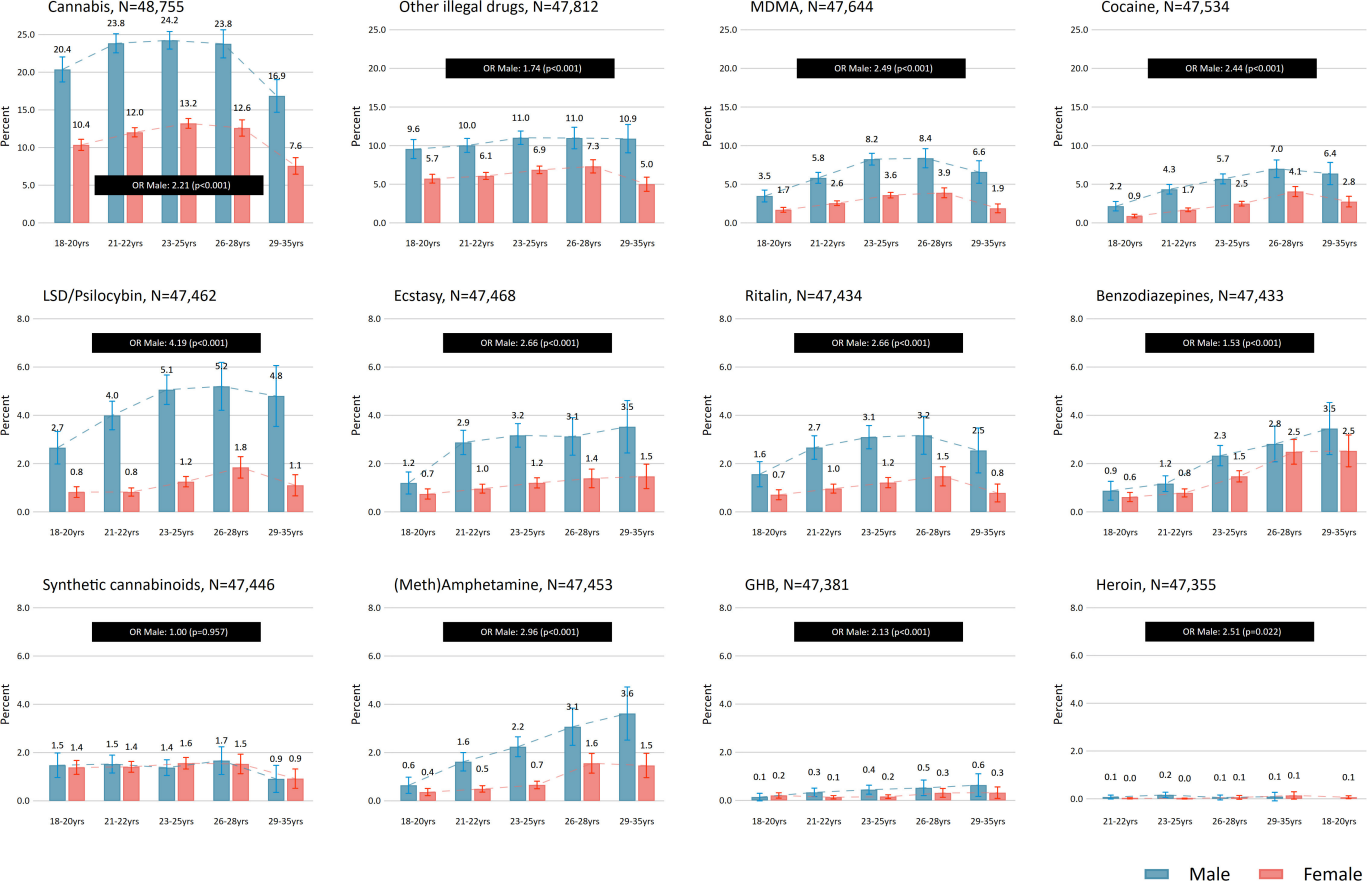
Last 12-months drug use (at least once) across gender and age. Overall gender difference presented as odds ratios (OR).

**Figure 4:**
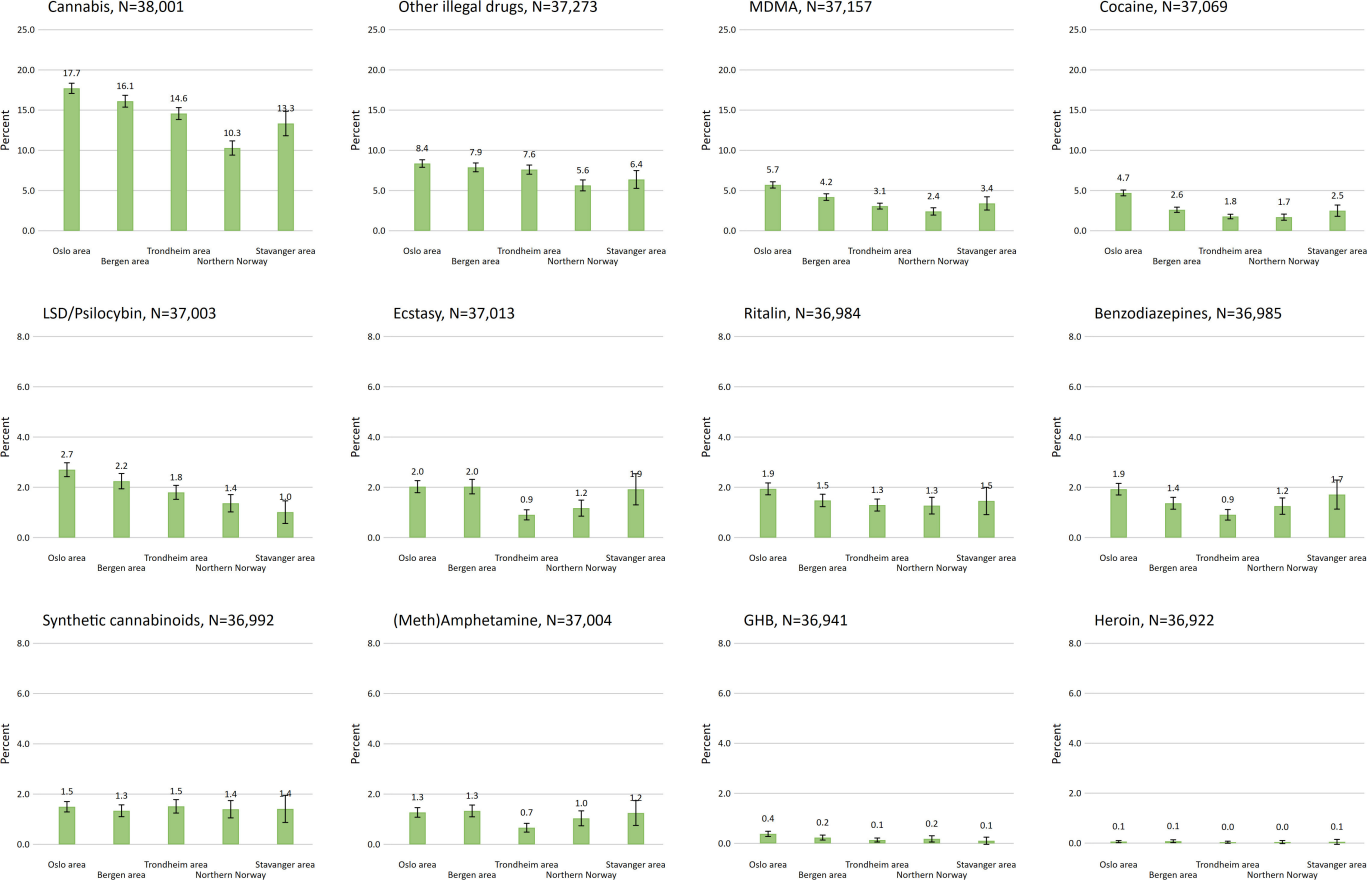
Last 12-months drug use (at least once) across regions. Adjusted for age and gender.

The age and gender distribution of the eligible 2018-sample was calculated. For the specific drug types, we calculated the frequency of drug use last 12-months based on categories described above using logistic regression models (Figure 2). Next, the percentages of specific illicit drug use across age groups and gender was calculated with 95% confidence intervals (CI) (Figure 3, Table 1). Associations between age and each specific drug types were tested for non-linearity and potential age × gender-interactions using likelihood-ratio tests (LR-tests). The age- and gender-adjusted proportion for specific drugs across geographic location was estimated (Figure 4). For the analysis across geographic location we used logistic regression adjusted for age and gender, and the margins function in Stata 15 to estimate the expected probability (percentage) of the individual drug use variables while averaging out the demographic characteristics. Also, only the largest study locations in Norway were included for the analyses across locations, yielding a maximum of *N* = 38,052 eligible participants. For all analyses an α of 0.05 was considered statistically significant, with the exception of comparisons with overall proportion for specific drugs in Table 1 (α = 0.001).

**Table 1:** Type of drug use last 12-months across gender and age groups (Norway 2018).

**Drug**	**Overall**	**Female**	**Male**	**18–20 yrs**	**21–22 yrs**	**23–25 yrs**	**26–28 yrs**	**29–35 yrs**
Cannabis (*N =* 48,755)	15.2 (14.9-15.6)	**11.8 (11.5–12.2)**	**22.9 (22.2–23.6)**	**12.9 (12.2–13.7)**	15.4 (14.8–16.0)	**16.8 (16.3–17.4)**	16.6 (15.6–17.5)	**10.7 (9.7–11.8)**
Other illegal drugs (*N =* 47,812)	7.6 (7.3–7.8)	**6.3 (6.1–6.6)**	**10.5 (10.0–11.0)**	**6.7 (6.2–7.2)**	7.2 (6.8–7.6)	**8.2 (7.8–8.6)**	8.6 (7.9–9.4)	7.0 (6.1–7.9)
MDMA (*N =* 47,644)	4.0 (3.8–4.2)	**2.8 (2.6–3.0)**	**6.7 (6.3–7.1)**	**2.1 (1.8–2.4)**	**3.5 (3.2–3.8)**	**5.1 (4.8–5.5)**	**5.5 (4.9–6.1)**	3.5 (2.8–4.1)
Cocaine (*N =* 47,534)	3.0 (2.8–3.1)	**2.1 (2.0–2.3)**	**5.0 (4.6–5.4)**	**1.2 (1.0–1.4)**	**2.4 (2.2–2.7)**	**3.5 (3.2–3.8)**	**5.1 (4.5–5.7)**	4.0 (3.3–4.6)
LSD/Psilocybin (*N =* 47,462)	2.1 (2.0–2.2)	**1.1 (1.0–1.2)**	**4.4 (4.0–4.7)**	**1.3 (1.1–1.5)**	**1.7 (1.5–1.9)**	**2.5 (2.2–2.7)**	**3.0 (2.6–3.5)**	2.3 (1.8–2.9)
Ecstasy (*N =* 47,468)	1.6 (1.5–1.7)	**1.1 (1.0–1.2)**	**2.8 (2.5–3.1)**	**0.9 (0.7–1.1)**	1.5 (1.3–1.7)	1.8 (1.6–2.1)	2.0 (1.6–2.4)	2.2 (1.7–2.7)
Ritalin (*N =* 47,434)	1.5 (1.4–1.6)	**1.0 (0.9–1.1)**	**2.7 (2.4–3.0)**	**0.9 (0.7–1.1)**	1.4 (1.3–1.6)	1.8 (1.6–2.0)	2.1 (1.7–2.4)	1.4 (1.0–1.8)
Benzodiazepines (*N =* 47,433)	1.5 (1.4–1.6)	**1.3 (1.1–1.4)**	**1.9 (1.7–2.1)**	**0.7 (0.5–0.9)**	**0.9 (0.7–1.0)**	**1.7 (1.5–2.0)**	**2.6 (2.2–3.0)**	**2.8 (2.3–3.4)**
Synthetic cannabinoids (*N =* 47,446)	1.4 (1.3–1.5)	1.4 (1.3–1.6)	1.4 (1.2–1.6)	1.4 (1.2–1.7)	1.4 (1.3–1.6)	1.5 (1.3–1.7)	1.6 (1.2–1.9)	0.9 (0.6–1.2)
(Meth)Amphetamine (*N =* 47,453)	1.1 (1.0–1.2)	**0.7 (0.6–0.8)**	**2.0 (1.8–2.3)**	**0.4 (0.3–0.6)**	**0.8 (0.7–1.0)**	1.2 (1.0–1.3)	**2.1 (1.7–2.5)**	**2.2 (1.7–2.7)**
GHB (*N =* 47,381)	0.2 (0.2–0.3)	**0.2 (0.1–0.2)**	**0.4 (0.3–0.5)**	0.2 (0.1–0.3)	0.2 (0.1–0.3)	0.2 (0.2–0.3)	0.4 (0.2–0.6)	0.4 (0.2–0.7)
Heroin (*N =* 47,355)	0.1 (0.0–0.1)	0.0 (0.0–0.1)	0.1 (0.0–0.1)	0.0 (0.0–0.1)	0.0 (0.0–0.1)	0.1 (0.0–0.1)	0.1 (−0.0–0.1)	0.1 (0.0–0.2)

Proportions and 95% confidence intervals.

Bold indicates statistical deviance from overall proportion (p < 0.001).

*N varies for rows due to missing data*.

### Handling of Missing Information (SHoT2018)

For age, 712 (1.4%) had missing information, while 218 (0.4%) lacked information about gender. For the question “Have you ever tried other drugs?,” 366 (0.7%) had missing information. Those with missing information on these three questions were excluded for further analyses (*N* = 1,226; 2.45%). Also, those answering affirmative on use of the non-existing drug ‘Relevin' (*N* = 10; 0.02%) were excluded. Those eligible for further analyses were *N* = 48,818, and in order to retain the maximum number of respondents for each drug, we used all valid responses for each drug category. The number of missing responses varied from most for heroin (*N* = 1,464; 3.0%) to least for cannabis (*N* = 63; 0.1%). It can be argued that it is most likely that those not answering specific drug types have not tried the specific drug during the last 12 months, yielding overall changes in the use estimates ranging from 0.16% (other illegal drugs) to <0.01% (heroin).

The reporting of the present study followed the STROBE guidelines (36).

## Results

### Descriptive Characteristics

In the eligible 2018-sample (*N* = 48,818) for this study, 30.7% were males, and the mean age was 23.3 (standard deviation 3.3). For the trend analyses, 31.7% were males, and ≈20% were aged 18–20 years, ≈60% were aged between 21 and 25 years, and ≈20% were aged 26 or more.

### Trends in Having Ever Tried Illicit Drugs (2010, 2014, 2018)

Overall, there was no difference between 2010 and 2014 in the reported use of drugs ever (22.8 vs. 21.9%, OR: 0.95, *p* = 0.189), but there was a significant increase from 2014 to 2018 (21.9 vs. 27.9%, OR: 1.32, *p* < 0.001). Gender-specific pairwise comparisons indicated no differences between 2010 and 2014 among males and a slight decline among females. Between 2014 and 2018 both the proportion for males and females increased (Figure 1, all *p*s < 0.001).

### Overall Drug Use (SHoT2018)

The mean age of the 2018-sample was 23.2 (standard deviation 3.3), and 69.3% were female.

Among the 48,818 eligible participants, 27.6% (*N* = 13,472) reported that they had tried to use drugs ever. In the past year, 18.6% (females 15.0%, males 26.8%) reported any use, and 11.1% (8.8% of females, and 16.3% of males) reported use of two or more types of drugs. In both cases males were more likely to report use last year (*p* < 0.001). The most commonly used drug type during the last 12 months was cannabis (15.2%), followed by MDMA (4.0%), cocaine (3.0%) and LSD/psilocybin (2.1%) (Table 1). For the other drug types, reported use was <2%, and GHB (0.3%) and heroin (0.1%) were the least used drugs. Figure 2 gives an overview of the frequency of use across drug types. For cannabis use, 4.7% reported use 5 times or more during the last year, while the corresponding number for MDMA was 0.6%. For all other specific types of illicit drug, use 5 times or more was reported by <0.5% of the sample, while use 5 times or more of other unspecified drugs were reported by 1.7%.

### Drug Use by Age and Gender (2018)

Having ever tried drugs was associated with age (OR-trend (average OR across levels): 1.34 per year, *p* < 0.001), where 17.7% of those aged 18–20 years reported having tried an illicit drug, compared to 38.5% in the age group 29–35 years. Overall, use of specific drugs last 12 months was associated with age for all types of illicit drugs except use of synthetic cannabinoids (*p* = 0.461 for linear term) and heroin (*p* = 0.054 for linear term) (Figure 3, Table 1). For use of GHB (OR-trend: 1.30, *p* = 0.002) and (meth)amphetamine (OR-trend: 1.50, *p* < 0.001) there was a linear relationship with age. For the remaining types of drugs, there was a non-linear, inverted U-shaped relationship with age (LR-test *p*-values ranging from *p* = 0.007 to *p* < 0.001).

Having ever tried drugs was more commonly reported by males than females (OR: 1.85, *p* < 0.001). Overall, males were more likely to report use of illicit drugs during the last 12 months (all *p* < 0.05), with the exception of synthetic cannabinoids (*p* = 0.912) (Figure 3, Table 1). The largest difference between genders were found for LSD/psilocybin (OR: 4.16) and the smallest difference for benzodiazepines (OR: 1.54). Males were also more likely to report drug use across all age-groups for cannabis, cocaine, Ritalin®, ecstasy, MDMA and LSD/psilocybin, as well as for unspecified drugs (all *p*-values < 0.05). Furthermore, gender differences were observed for all age groups except among 18–20 year olds for (meth)amphetamine use. For GHB and benzodiazepines, gender differences were observed for ages 21–22 and 23–25 years only. No interaction between gender and age for any type of drug use was observed (LR-test *p*-values ranging from 0.187–0.855).

### Drug Use by Location (2018)

Having ever tried drugs was associated with geographic location, with the highest proportion in the Oslo area (30.9%; 95%CI: 30.1–31.7) and the lowest proportion in Northern Norway (22.0%; 95%CI: 20.9–23.3). There was no statistical difference in ever having tried drugs between the Stavanger, Trondheim, and Bergen area. For specific drug use last 12 months, there was little geographic variation in the reported use of synthetic cannabinoids, heroin and (meth)amphetamine (Figure 4). For all other drugs, we observed differences across regions. The largest geographic differences (maximum vs. minimum) was observed for cannabis [7.4 percentage points (pp)], MDMA (3.3 pp), cocaine (3.0 pp), LSD/psilocybin (1.7 pp), ecstasy (1.1 pp), benzodiazepines (1.0 pp), and other illegal drugs (2.8 pp), while the differences was less pronounced for the remaining drug types (GHB and Ritalin®). The Oslo area reported more frequently any use of cannabis, cocaine, GHB, Ritalin®, MDMA, LSD/psilocybin, and other unspecified drugs during the last 12 months compared to the rest of the geographic areas. The Northern Norway reported the lowest frequency of any drug use in general. Despite difference in the use of specific drugs between locations, estimates of intraclass correlations were low (all ICC < 0.05) indicating low drug use resemblance based on grouping by study location.

## Discussion

The present study from a large sample of Norwegian full-time higher education students aged 18–35 years suggests that illicit drug use is becoming an important public health issue in this population. While we found that the rates of having tried illicit drugs had remained stable from 2010 to 2014, there was a significant increase during the time frame from 2014 to 2018. This increase in having ever tried illicit drugs was evident for both males and females.

We also found that any past year illicit drug use was high in the SHoT2018-dataset compared with the two earlier surveys. The rates of illicit drug use in our sample exceeds the rates in a relatively recent Norwegian general population sample aged 16–30 years, in which 14.1% of males and 6.4% of females reported past year cannabis use (37), compared with 22.9 and 11.9% in our sample, respectively. Similarly, comparing our data with the report from Sandøy (37), the male students exceed their counterparts in the general population on past year use of cocaine (5.0 vs. 3.5%), amphetamines (3.9 vs. 1.6%), and MDMA/ecstasy (7.0 vs. 1.3%). These findings are not surprising, and lend support to previous contributions that points to a higher illicit drug use among higher education students, compared with young adults in the general population (1, 10). On the other hand, 40% of respondents in a Norwegian nightlife setting reported past year cannabis use (27), which in turn far exceeds the estimated past year cannabis use in the current sample of students in higher education.

Moreover, the rates of past year use were significantly higher for males for all types of illicit drugs, except synthetic cannabinoids and heroin (where numbers were very small overall). These findings lend support to previous studies from student populations that also report higher illicit drug use among males [e.g., (31)]. We found geographical differences in proportions of past year drug use, in which students in the Oslo area had either the highest or as high past year use across all substances compared with other areas. Students in Northern Norway were in the lower end of the past year use for most substances. While any lifetime illicit drug use showed a linear increase by age, past year illicit drug use peaked in the age span between 23 and 28 years of age for most types of drugs, with some exceptions.

In support of a range of previous publications (1, 5, 19, 20), cannabis was by far the most commonly used illicit drug across genders among the higher education students. Cannabis use showed an inverted U-curve across age, peaking in the age span from 23 to 28 years of age. The estimates of past year cannabis use fall within the higher range of prevalence rates in higher education students from other countries. While a UK study of university students reported that 31% of male and 14% of female students had used cannabis during the past year (24), a study of Swedish University students reported that 12% of male and 7% of female students reported past year cannabis use, with marked differences in rates across geographical areas (19).

The relatively high proportions of cannabis use among the Norwegian higher education students is worrying. A US study of 17–20 year old college students reported that approximately one out of four of all past-year cannabis users fulfilled criteria for a cannabis use disorder (CUD) (21). In addition, Caldeira et al. found that students reporting five or more times with cannabis use past year, concentration problems (40%), driving while high (19%), and missing class (14%) were the most common cannabis-related problems, even among those who endorsed no CUD criteria (21). In the present study, ~5% of the sample reported at-risk (5 times or more) past year cannabis use. In addition, a study by Lac and Luk (22) reported that cannabis use significantly and longitudinally prompted lower initiative and persistence. Hence, due to the high rates of past year cannabis use in our sample, interventions that target cannabis use in student settings are needed.

Past year use was also relatively high in our sample for MDMA, cocaine, and LSD/psilocybin. An inverted U-curve by age was found for these substances, peaking between 23 and 28 years of age. The relatively common use of MDMA in our sample, particularly among male students (6.7% compared with 2.8% among females), is an unexpected finding. Adding to the reported MDMA use, 2.8% of male and 1.1% of female students in our sample reported past year use of ecstasy (in which MDMA is the main ingredient). The total aggregated use of MDMA/ecstasy in our sample was 4.2%; 7.0% for male and 3.0% for female students. In comparison, a UK study of university students reported that 10.5% of male and 4.5% of female students had used ecstasy the past year (24). The use of MDMA/ecstasy was also among the most commonly reported drugs in a recent study from a Norwegian nightlife setting in Oslo (27), which however reported cocaine as the second most used drug after cannabis. Interestingly, a recent publication documented the re-emergence of MDMA as an important psychoactive substance in Norwegian nightlife scenes (38). A perceived distinction between MDMA (in crystal or powder form) and ecstasy (in pill form) may have contributed to this re-emergence, favoring MDMA as a “safer drug,” even though the main active ingredient in both drugs is MDMA (38). The risks associated with the use of MDMA/ecstasy are disputed (39–41); however the perceived safety of MDMA may be an important target for intervention in student populations.

Cocaine use was also quite common in the student sample, particularly among male students. The proportions of past year cocaine use were far below the estimates from a US study on college students aged ~18–22 years of age (i.e., from first to fourth year of college) (42). The authors reported that past year cocaine use increased gradually over time, from 4% the first year to 10% the fourth year, with little gender differences in rates (42). In comparison, we found that rates of cocaine use increased from 1.2% at age 18–20 to 5.1% at age 26–28, after which the rates decreased. A UK study reported that 8.8% of male and 3.8% of female university students had used cocaine past year (24), which is also somewhat higher than in the present study. Interestingly, an Italian study of university students even reported that cocaine was the second most prevalent drug (after cannabis) in relation to lifetime use, reported by 13% of the sample, which was deemed as surprisingly high (20). Similarly, cocaine was the second most commonly used illicit drug in a Norwegian nightlife setting (27). On the other hand, a Swedish study of university students aged 16–25 years reported a lower rate of past year cocaine use (0.7%) compared with our sample. Thus, cocaine use is clearly prevalent in higher education students in some countries, something which was confirmed in our study, notably among males.

LSD/psilocybin was also among the more commonly used substances in our sample, with a rather large gender difference (4.4% among males; 1.1% among females). In a recent study of UK University students, it was reported that 4.6% of male and 0.8% of female students had used LSD, while 5.4% of male and 2.4% of female students had used magic mushrooms (i.e., psilocybin), during the past year (24). As the UK study did not use an aggregated measure LSD/psilocybin, their findings are difficult to compare directly to results from the present study. Interestingly, the past year use of LSD in the general young adult Norwegian population was deemed as negligible small (37). On the other hand, past year LSD use was reported by 4% of male and 2% of female young adults in a Norwegian nightlife setting (27), indicating that LSD is being used in the Norwegian population.

Past year use for (meth)amphetamine (2.1% among males; 0.7% among females) and Ritalin® used without prescription (2.7% among males; 1.0% among females) were relatively modest in our sample. The total use of amphetamines in our sample was 2.2%, and 3.9% in male and 1.5% in female students. Whereas, (meth)amphetamine showed a linear trend with increased use with age, the use of Ritalin® followed an inverted U-shape with its peak in the age span from 23 to 28 years of age. A UK study on university students found a similar proportion of amphetamine use (4.5% among males; 2.4% among females) (24). Amphetamine use among higher education students has specifically received scientific attention as a potential drug used for neuro-enhancement, i.e., the attempt to improve cognitive performance (43), in addition to other performance-enhancing substances (44). However, only 0.4% of university students in Swiss study reported use of amphetamine for this purpose (45). The present study did not explore motives for substance use, and we may therefore not conclude on the extent of which enhancement motives constitute a significant motive for illicit drug use in general, as well as amphetamines in specific, in our sample. This could be of interest to further studies on students and substance use.

The use of synthetic cannabinoids was very low in our sample. These substances constitute a large group of drugs that have an effect similar to cannabis, but many of these substances are considered to be more potent (46) and are related to serious psychiatric and medical conditions and can even lead to death (47). This rate was similar across genders, with no evidence of geographical differences or association with age. As this drug is frequently marketed over the internet as legal and harmless (46), it may be an important drug to further monitor in a student setting.

Interestingly, a recent study using the same datasets highlighted that alcohol use among Norwegian students have remained relatively stable during the time frame from 2010 to 2018 (3). Also of note, according to a large national survey on Norwegian upper secondary school youth (aged 16–19 years), alcohol use have remained relatively stable during the past 4 years, while cannabis use have increased (48). As such, it appears that the increased substance use among Norwegian higher education students may be specific to illicit drug use, resonating with a tendency that is also found in the general, somewhat younger, Norwegian population.

Potential mechanisms for changing trends in substance use include changes in availability, harm perception, and attitudes toward use (25), and positive attitudes to illicit drug use is a particularly strong risk factor for high-frequency use (49). As the present study do not have available data on potential changes in these aspects, interpretation of reasons for the increase in lifetime illicit drug use is hampered. Thus, we recommend that future studies evaluate the extent to which there has been an increase of positive attitudes as well as reduced perceptions of harm related to illicit drug use among students in higher education, and to what extent these factors may explain the observed increase in lifetime illicit drug use. Aggregate media coverage has previously found to be related to young people's illicit drug use (50, 51) and could therefore be an important factor to be evaluated in relation to potential changes of attitudes and harm perception toward illicit drug use.

Our findings add to previous results that demonstrate high levels of substance use among Norwegian students (3) and underscore the need for substance use prevention in this population. A report authored by the Norwegian Institute of Public Health (52) summarizes the national actions undertaken specifically to reduce substance use among students in higher education in the period from 2007 to 2016. A stronger coordination of this effort as well as utilization of interventions that are available for effect evaluations is recommended, for example by using other student locations as control groups when specific measures are implemented at a target student location (52). The SHoT-survey is specifically highlighted in the report as an important source for knowledge on the development of substance use among Norwegian students in this respect.

### Strengths and Limitations

A notable strength of the present study was the combination of a very large sample size, and the inclusion of an array of different types of illicit drugs as well as frequency of past year use. The inclusion of the “fake drug,” Relevin is also a strength, as it served as an indirect test of how many students that were giving false positive answers. The number of positive responses on the fake drug was negligible small. Although illicit drug use potentially could be underreported in studies based on self-report due to the stigmatized and undesirable behavior in question (53), it should be noted that the convergent validity of self-reported illicit drug use and urine tests is satisfactory among university students (54). Across the three waves of the SHOT-survey, some changes were made to the general content and sampling design. In general, the scope and themes covered expanded from 2010 to 2018, but the questions relevant for the present study remained unchanged. With regards to sampling design, the number of welfare organizations and institutions increased between 2010 and 2018. In a recently published paper explicitly investigating comparability between the waves, however, only small differences were found between the waves. This finding suggests that the three samples are comparable despite an expanding sampling frame with time (33). The main limitation of the present study was the response rate of 31% in the SHoT2018 survey, as well at the even lower response rate in 2010 and 2014 (23–29%). The low response rate raises questions regarding the representativeness of our sample, as well as the generalizability of the results, and we advise that interpretation of our findings is made with caution. However, the dataset used in this study comprise the largest dataset on higher education student illicit drug use in Norway, and no better data sources are available. The representativity issue is actualized in the 69% female composition of the SHoT2018 sample (3), which was larger than the female composition (58%) of the invited students. Thus, our estimates of overall illicit drug use may be biased, while associations between illicit drug use and age, gender, and location are probably valid. In addition, all eligible students were included at each wave. Thus, it is possible (and likely) that some of the same students were recruited across at least two waves. We were, however, not able to discern those participating multiple times and those who have only participated once, due to anonymous participation in 2010 and 2014. Another limitation was that no questions were asked about the timing of initiation of drug use or duration of use, and our findings are thus not likely to measure trends in patterns of illicit drug use, i.e. how illicit drug use have started and how it has been used over a longer period of time. Finally, the SHoT-surveys were conducted in different times of the year, something that may also be a limitation (for details, see 3).

## Conclusions

The current study presents results from a large Norwegian sample of higher education students and demonstrated that use of some illicit drugs was fairly common in this population. The rates of having tried illicit drugs have increased during the period 2010–2018, indicating that a substantial proportion of the students have tried illicit drugs—particularly among male students. Regarding past year use, cannabis was by far the most commonly reported drug across gender and age groups, while MDMA, cocaine, and LSD/psilocybin was also quite common. These findings demonstrate that illicit drug use can constitute an important public health issue among students in higher education, and that information regarding the different substances should be provided to students.

## Data Availability Statement

Publicly available datasets were analyzed in this study. The SHoT dataset is administrated by the NIPH. Approval from a Norwegian regional committee for medical and health research ethics [https://helseforskning.etikkom.no] is a pre-requirement. Guidelines for access to SHoT data are found at [https://www.fhi.no/en/more/access-to-data].

## Ethics Statement

The studies involving human participants were reviewed and approved by The Regional Committee for Medical and Health Research Ethics in Western Norway (no. 2017/1176; the SHoT2018-survey) and the data protection officer for research at the Norwegian Centre for Research Data (the SHoT2010- and SHoT2014-surveys). The patients/participants provided their written informed consent to participate in this study.

## Author Contributions

OH conducted preliminary statistical analyses, literature search, and wrote the introduction and discussion sections. JS finalized the statistical analyses and wrote the methods and results sections. BS and K-JL were responsible for conception and design of the SHoT study, and K-JL obtained funding. All authors were involved in interpreting the results, critially revised and contributed to the manuscript, and read the final manuscript.

## Conflict of Interest

The authors declare that the research was conducted in the absence of any commercial or financial relationships that could be construed as a potential conflict of interest.
